# Measuring visual electrophysiological responses in individuals with low-functioning autism: a feasibility and pilot study

**DOI:** 10.1186/s40814-021-00960-7

**Published:** 2022-01-14

**Authors:** Kyongje Sung, Hanna Glazer, Jessica O’Grady, Mindy L. McEntee, Laura Bosley, Dana Boatman Reich, Barry Gordon

**Affiliations:** 1 Department of Neurology, Johns Hopkins University School of Medicine, 600 N. Wolfe Street, Meyer 2-147, Baltimore, Maryland 21287 USA; 2 Counseling, Special Education, and Neuroscience Division, Emirates College for Advanced Education, Abu Dhabi, United Arab Emirates; 3 College of Health Solutions, Arizona State University, Phoenix, Arizona USA; 4 Cognitive Science Department, Johns Hopkins University, Baltimore, Maryland USA

**Keywords:** Autism, Visual evoked potentials, EEG alpha spectral power, Low-functioning autism

## Abstract

**Background:**

Although visual abnormalities are considered common in individuals with autism spectrum disorders, the associated electrophysiological markers have remained elusive. One impediment has been that methodological challenges often preclude testing individuals with low-functioning autism (LFA).

**Methods:**

In this feasibility and pilot study, we tested a hybrid visual evoked potential paradigm tailored to individuals with LFA that combines passively presented visual stimuli to elicit scalp-recorded evoked responses with a behavioral paradigm to maintain visual attention. We conducted a pilot study to explore differences in visual evoked response patterns across three groups: individuals with LFA, with high-functioning autism (HFA), and with typical development.

**Results:**

All participants with LFA met criteria for study feasibility by completing the recordings and producing measurable cortical evoked waveform responses. The LFA group had longer (delayed) cortical response latencies on average as compared with the HFA and typical development groups. We also observed group differences in visually induced alpha spectral power: the LFA group showed little to no prestimulus alpha activity in contrast to the HFA and typical development groups that showed increased prestimulus alpha activity. This observation was confirmed by the bootstrapped confidence intervals, suggesting that the absence of prestimulus alpha power may be a potential electrophysiological marker of LFA.

**Conclusion:**

Our results confirm the utility of tailoring visual electrophysiology paradigms to individuals with LFA in order to facilitate inclusion of individuals across the autism spectrum in studies of visual processing.

**Supplementary Information:**

The online version contains supplementary material available at 10.1186/s40814-021-00960-7.

## Key messages regarding feasibility

- What uncertainties exist regarding feasibility? Individuals with LFA are often unable to perform behavioral paradigms or attend consistently to stimuli used in visual evoked studies.
- What are the main feasibility findings? Study completion and adherence rates were 100% for participants with LFA who also showed delayed visual evoked response latencies and decreased prestimulus alpha power compared to participants with high-functioning autism and typical development.
- What are the implications of the feasibility findings? Our findings confirm the feasibility and potential utility of including individuals with LFA in evoked potential studies of visual processing in autism.

## Background

Visual processing abnormalities are considered common in autism spectrum disorder (ASD), a complex neurodevelopmental disorder characterized by impaired social interaction and communication and stereotyped behaviors and interests [1]. Despite considerable research, there are no known electrophysiological markers of atypical visual processing in individuals with ASD. One impediment has been the challenge of testing individuals from across the ASD spectrum and, in particular, individuals with low-functioning autism (LFA) who are often unable to adhere to the behavioral demands of traditional visual evoked potential paradigms or behavioral and neuroimaging studies in general [2, 3]. Individuals with LFA, also referred to as ASD level-3 based on DSM-5 criteria [1], typically have more severe ASD features, including minimal verbal skills and inability to function independently in daily living. LFA is one of two widely used classifications for individuals with ASD. The other is high-functioning autism (HFA), also referred to as ASD level-1 [1], which refers to individuals who are moderately to highly verbal and require minimal or no support for daily living.

Evoked potential studies based on passive stimulus paradigms have been used successfully to investigate sensory processing across the autism spectrum, including auditory and somatosensory processing [4–7]. However, passive visual stimulus paradigms can be challenging to implement with individuals with LFA because they must be visually attending whenever stimuli are presented in order to elicit evoked responses.

To address the methodological challenges of conducting visual evoked potential studies with individuals with LFA, we explored the feasibility of using a hybrid visual paradigm that combines passively presented stimuli to elicit evoked potentials with a different set of stimuli in a visual task to ensure participants are visually attending when passive stimuli are presented. Feasibility was assessed based on study completion rates and quality of recordings. We then conducted a pilot study that included two additional groups—individuals with high-functioning autism (HFA) and individuals with typical development (TD)—to investigate potential patterns of cortical visual activity that may be specific to individuals with LFA, based on evoked responses and spectral (oscillatory) power measurements.

## Method

### Study participants

A total of 18 individuals, ages 17–48 years (15 males, 3 females), participated in the study. Participants were assigned to one of three groups (Table 1), each with six participants (5 males, 1 female): an LFA group, an HFA group, and a TD group. The diagnosis of autism was based on physician diagnoses, as confirmed by participants’ medical records and DSM-5 criteria [1], and scores of ≥10 on the Autism Diagnostic Observation Schedule Second Edition (ADOS-2) [8]. The mean age of participants with ASD was 29.8 years ±9.0; the mean age of participants with TD was 26.0 years ±4.8.

**Table 1:** Participant Demographics

Participant	Sex	Age (yrs)	Neuropsychological Testing	Speech-Language Abilities	Medications (daily dosage)
P1_LFA	M	17	ADOS Module 1 (composite score 21); PPVT-IV-B (score 20)†	Nonverbal	Vitamins; digestive enzymes
P2_LFA	M	25	ADOS Module 2 (composite score 23); KBIT-2 (composite IQ 44); PPVT-IV-B (score 36)	Minimally verbal	Risperidone (1mg); Cetirizine (10mg); Lithium (1500 mg)
P3_LFA	M	39	ADOS Module 4 (composite score 22); KBIT-2 (composite IQ 61); PPVT-IV-B (score 58)	Minimally verbal: single words only; monotonic	Sertraline (50mg), Clomipramine (50mg)
P4_LFA	M	48	ADOS Module 4 (composite score 19); KBIT-2 (composite IQ 112); PPVT-IV-B (score 94)	Minimally verbal: single words only; aversion to /pl/ and other speech sounds	Sertraline (100mg); Amlodipine (5mg)
P5_LFA	M	28	ADOS Module 4 (composite score 18); KBIT-2 (composite IQ 66); PPVT-IV-B (score 62)	Minimally verbal	Loratadine (10mg)
P6_LFA	F	26	ADOS Module 4 (composite score 19); KBIT-2 (composite IQ 64); PPVT-IV-B (score 45)	Minimally verbal	NA
P7_HFA	M	26	ADOS Module 4 (composite score 14); KBIT-2 (composite IQ 77); PPVT-IV-B (score 71)	Verbal	NA
P8_HFA	M	26	ADOS Module 4 (composite score 10); KBIT-2 (composite IQ 129); PPVT-IV-B (score 136)	Verbal	Duloxetine (30mg)
P9_HFA	F	21	ADOS Module 4 (composite score 14); KBIT-2 (composite IQ 80); PPVT-IV-B (score 88)	Verbal	NA
P10_HFA	M	45	ADOS Module 4 (composite score 10); KBIT-2 (composite IQ 144); PPVT-IV-B (score 137)	Verbal	Ropinirole (dosage undisclosed)
P11_HFA	M	30	ADOS Module 4 (composite score 10); KBIT-2 (composite IQ 89); PPVT-IV-B (score 98)	Verbal	NA
P12_HFA	M	27	ADOS Module 4 (composite score 18); KBIT-2 (composite IQ 103); PPVT-IV-B (score 116)	Verbal	Sertraline (200mg); Quetiapine (200mg)
P13_TD	M	26	NA	Verbal	NA
P14_TD	M	25	NA	Verbal	NA
P15_TD	M	28	NA	Verbal	NA
P16_TD	M	22	NA	Verbal	NA
P17_TD	F	20	NA	Verbal	NA
P18_TD	M	35	NA	Verbal	NA

LFA: Low-Functioning Autism; HFA: High-functioning Autism; TD: Typical development. ADOS: Autism Diagnostic Observation Schedule (2^nd^ ed); PPVT-III (standard score): Peabody Picture Vocabulary Test-III; PPVT-IV-B (standard score): Peabody Picture Vocabulary Test-IV-form B; KBIT-2: Kaufman Brief Intelligence Test-2; All tests were administered at the time of study participation

†KBIT-2 test was not administered

Participants with ASD were classified as LFA or HFA based on the level of support required for daily living and on verbal communication abilities. Individuals classified as LFA required full support for daily living and were either nonverbal or minimally verbal; individuals with HFA required little or no support for daily living and demonstrated good verbal language abilities, including spontaneous speech production. To further assess language and cognitive function, all ASD participants completed the Peabody Picture Vocabulary Test and all but one ASD participant were also administered the Kaufman Brief Intelligence Test-2 (Table 1). The one exception was a participant with LFA (P1_LFA) who was unable to follow test directions to perform the Kaufman Brief Intelligence Test-2.

ASD participants were recruited through educational programs and a local institution that provides day services to individuals with autism and other developmental disorders. ASD and TD individuals were also recruited through newspaper advertisements and fliers posted at regional academic centers. Individuals were excluded from participation if they had a history of neurologic disease (e.g., epilepsy) or substance abuse, vision deficits, or demonstrated excessive movements or an inability to tolerate the electrode net used for the electrophysiology recordings. A total of nine individuals with LFA was originally recruited of whom three were excluded due to excessive movement and/or inability to wear the electrode net. All participants had normal or corrected-to-normal vision based on parental/caregiver reports (LFA) or self-reports (TD, HFA). Informed written consent was obtained from, or on behalf of, all participants: HFA and TD participants provided written informed consent; parents, guardians, or caregivers provided consent for LFA participants. All participants were paid $15 per hour for their participation. All procedures and consent processes were approved by the Institutional Review Board of the Johns Hopkins Medical Institutions.

### Experimental procedures

#### Experimental set-up

The experimental set-up was the same for all participants. Each participant was tested individually in a quiet room where they were seated in front of a 34.5 x 27.5 cm LCD computer screen (1280 x 1024 pixel resolution, 60 Hz temporal resolution) with attached computer mouse. Viewing distance from the screen was approximately 50 cm; room lighting was dimmed to enhance visualization of the screen. Data collection was conducted by pairs of trained research assistants (HG, MM, LB) who were experienced in conducting electrophysiology studies with individuals with autism. A senior researcher (KS) with expertise in electrophysiology and signal processing was also present during the recording sessions to assist with the experimental set-up and technical issues and to monitor the recordings.

Based on prior observation that individuals with LFA often show increased agitation in unfamiliar situations, we implemented two familiarization strategies. First, all participants with LFA underwent one or two sessions of net tolerance training during the month prior to the study to familiarize them with wearing the electrode nets. Second, a family member or caretaker was present during testing, and at least one member of each researcher pair was introduced to the participant prior to the date of the study. The participant’s family member or caregiver also helped determine when session breaks were needed during testing.

#### Experimental stimuli

The main visual stimulus was a static black-and-white checkerboard pattern that appeared as a brief flash (100-ms duration) on the computer screen. The checkerboards were full-screen patterns comprised of 1.83 cm^2^ squares. A second visual stimulus was a pair of shapes, a white square and a white circle both 8 cm in size (length/diameter), presented side-by-side in the center of the screen. The location of each shape on the left or right side varied randomly across trials. The luminance of the white- and black-colored stimuli on the screen was 140 cd/m^2^ and 0.3 cd/m^2^, respectively.

### Experimental task

Stimulus trials were presented consecutively with checkerboard trials interspersed randomly among shape-pair trials. The trial structure was the same for both stimuli. Each trial began with a blank (black) screen, lasting for 500 ms, followed by a white fixation cross in the middle of the screen for 1000 ms. This was followed by a second blank screen that lasted for 1200 ms, followed by presentation of either a checkerboard stimulus or a shape-pair stimulus. Each trial was followed by a 1000-ms blank screen. All stimuli were presented using E-prime software (version 2.0.8.90, Psychology Software Tools, Inc., Sharpsburg, Pennsylvania) running on a Windows XP PC (Dell OptiPlex 755, Dell Technologies, Round Rock, Texas).

The checkerboard trials were presented passively: no behavioral response was required and participants were not told about them beforehand. Passive presentation of the checkerboard trials controlled for individual differences in attentional states and behavioral performance across participants. The shape-pair trials served to encourage sustained visual attention to the computer screen by engaging participants in a behavioral task. Participants were instructed to respond on shape-pair trials by using the computer mouse to click on the circle shape. Participants with LFA were given verbal instructions, demonstrations, and practice trials before the session. Each pair of shapes remained on the screen until participants responded or until 10 s had elapsed. To ensure that participants moved the mouse to the target shape, the mouse pointer arrow returned to the bottom center of the screen before each trial. Participants’ behavioral responses were recorded online for analysis (accuracy, reaction time).

To minimize the time, the participants were required to sit still and to facilitate breaks during the session, and trials were grouped into five blocks of 108 trials each. Each block contained both visual stimuli in a 5:1 ratio of shape-pair trials to checkerboard trials. For two participants with LFA (P2 and P3), the number of stimulus blocks was increased to 10 (P2) and nine (P3) blocks of 72 trials. By both increasing the number of blocks and decreasing their length, participants could be given more frequent breaks while completing additional trials without extending the duration of the recording session.

Only checkerboard trials were included in the electrophysiology analysis. The total number of checkerboard trials presented to participants ranged from 108 to 140, depending on the number of stimulus blocks administered. For participants with LFA, the average number was 122 checkerboard trials. To verify that participants with LFA were attending to the computer screen when checkerboard stimuli were presented, two high-definition camcorders were used to video record each session: one recorded a frontal face view; the second recorded a rear view, including the computer screen (Sony HDR-CX360, Sony Corporation, NYC, New York).

#### Electrophysiology recording procedures

At the beginning of each recording session, a 256-channel HydroCel Geodesic Sensor electrode net that had been soaked in electrolyte solution (tap water with potassium chloride; 11,000mg/L) was fit onto each participant’s head. The electrode nets remained on for the entire session, including breaks.

Continuous EEG recordings were acquired at a sampling rate of 250 Hz, using a vertex electrode as the reference and an anti-aliasing filter cut-off frequency of 4 kHz. Electrode impedances were maintained below 50 kΩ. The recordings were acquired using a Geodesic EEG system, Net Amps 300 amplifier, and NetStation 4.3 software (Electrical Geodesics, Inc., Eugene, Oregon) on a Mac Pro computer (OS X version 10.6.8, Apple, Inc., Cupertino, California).

Each block of trials took approximately 9 min to complete. The session duration ranged from 45 min to 1 h, depending on the number of breaks required. For participants with LFA, several shorter sessions with longer breaks were scheduled on the same day to maintain adherence and increase the likelihood of acquiring recordings that were not contaminated by movement or other artifacts. The need for multiple shorter sessions was based on feedback from caregivers and informal evaluation of participants’ moods, behaviors, and willingness to follow directions. One participant with HFA returned for a second session because software difficulties resulted in early termination of the first session.

### Data analysis

#### Electrophysiology recordings

##### Signal preprocessing

Signal preprocessing was performed using MATLAB (v. R2018b; Mathworks) and the EEGLAB toolbox [v. 14.1.0b; http://sccn.ucsd.edu/eeglab/;^9^]. The continuous EEG signals were high-pass filtered at 0.1 Hz and low-pass filtered at 38 Hz (Hamming windowed sinc FIR filter). Channels with excessive noise or artifact were identified for exclusion based on the voltage histogram [−500, 500 μV; bin size 10]. Channels with voltages that deviated from the mean-normalized voltage by ±30μV were excluded and interpolated with a spherical electrode configuration. The EEG signals were re-referenced to an average reference and segmented into epochs using a 2-s window that included a prestimulus period of 1000 ms. Epochs with ocular, cardiac, or other artifacts were identified for rejection based on independent component analysis (ICA) implemented in EEGLAB toolbox [10]. Because the data were relatively short in length, especially for participants with LFA, the principal component analysis was used to improve ICA outcomes by reducing the high-dimensional, 256-channel data to a smaller set of 64 principal components that accounted for ≥ 97% of the original data. The dimension-reduced data were then decomposed into independent components. The ICA components and their topographic distributions were examined to identify EEG artifact for rejection. Following ICA artifact rejection, only epochs (trials) associated with the checkerboard stimulus were selected for the visual evoked potential and power spectrum analysis. All checkerboard trials were reviewed to eliminate trials with excessive movement artifact, as determined visually (KS) and confirmed by review of the video recordings. Independent review of the video recordings was performed (HG, MM, LB) to eliminate any checkerboard trials that were presented when participants were not attending to the computer screen. The average trial rejection rates for each of the three participant groups were 21% for TD, 19% for HFA, and 30% for LFA participants.

##### Visual evoked potentials (VEP)

For each participant and electrode channel, VEP responses to the checkerboard stimuli were computed by trial averaging in the time domain. To focus on early cortical evoked potentials, the duration of the original 2-s epochs was trimmed to 700 ms [−200, 500 ms]. Each epoch (trial) was then normalized to the prestimulus voltage by subtracting the mean prestimulus voltage [−200, 0 ms] from the poststimulus signal. VEPs were measured at the occipital midline electrode (Oz), where the largest VEPs and alpha oscillations were observed across all participants.

For each participant, peak response latency and amplitude measurements (base-to-peak) were derived for the P1-N1-P2 components in the 0–300 ms period following stimulus presentation. The P1 was identified as the largest positive waveform deflection between 45 and 70 ms after stimulus presentation; the N1 was the largest negative waveform following the P1, occurring between 70 and 130 ms; and the P2 was the next largest positive waveform occurring between 120 and 250 ms. The total number of checkerboard trials averaged for group VEPs was 494 (LFA), 407 (HFA), and 397 (TD). The average number of checkerboard trials for each group was 82.3±18.3 (LFA), 67.8±10.9 (HFA), and 66.2±8.2 (TD). Group-level VEPs were computed by averaging across participants within a group, and peak waveform measurements were derived using the same procedures implemented for individual waveform measurements.

##### Spectral power analysis

Time-frequency analysis was used to measure the overall power of cortical EEG oscillations and to identify event-related changes in spectral power (ERSP) with visual stimulation. Time-frequency analysis of single trials was performed using wavelet analysis (*newtimef* function, EEGLAB). To ensure the response time window was sufficiently long to capture the slower cortical EEG oscillations (<10 Hz), the original, 2-s epoched data was used. Time-frequency analyses were performed at both the individual and group levels.

To compute the overall time-frequency power spectrum, the power of 27 linearly spaced EEG frequencies from 4 to 30Hz was calculated using a wavelet time window of 1 s. This was done for all 256 channels to generate cortical topographical maps of the power distributions. The time window of the resulting power spectrum was then trimmed to −500 to 500 ms. The overall time-frequency power spectrum was plotted along with the log-transformed power spectrum density profile for the prestimulus and poststimulus periods. To further examine the cortical distribution of spectral power, topographical maps of the pre- and poststimulus spectral power were plotted across all electrode channels with interpolation (*topoplot* function, EEGLAB). ERSP was calculated for channel Oz by subtracting the mean prestimulus power of each frequency from the corresponding frequency occurring 0–500-ms poststimulus. We used non-parametric, Monte Carlo-based permutation testing methods in EEGLAB to assess the statistical significance of poststimulus changes in spectral power relative to baseline (prestimulus) at an alpha level of 0.05. To correct for multiple comparisons that can inflate type I error rates, we used the false discovery rate [11]. We also performed the power spectrum analysis at the individual level to compute bootstrapped confidence intervals for alpha and theta powers of prestimulus and poststimulus time windows.

##### Behavioral analysis

Although shape-pair trials were not included in the electrophysiology analysis, we analyzed participants’ behavioral response accuracy and latency to confirm comprehension of the task, adherence, and visual attention to the computer screen.

## Results

We first assessed the feasibility of using a hybrid visual electrophysiology paradigm to test individuals with LFA based on participant adherence and completion rates. All participants with LFA completed the testing and met adherence requirements: they remained seated, attended to the computer screen, and wore the electrode nets for the duration of the recording sessions, including breaks.

**Fig. 1:**
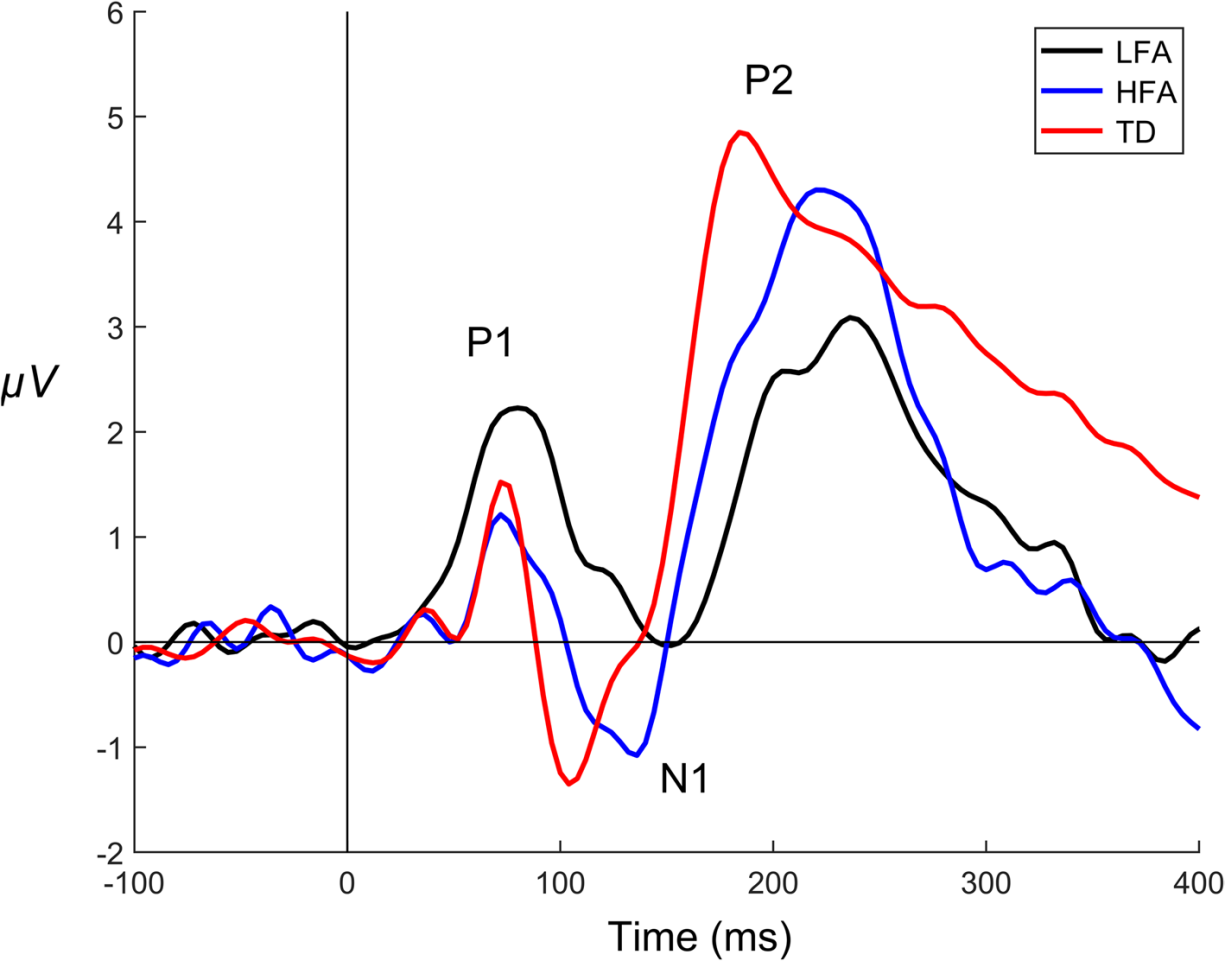
Visual evoked potential waveforms recorded from electrode Oz and averaged by participant group. LFA low-functioning autism, HFA high-functioning autism, TD typical development. Time is on the *x*-axis in milliseconds; amplitude is on the *y*-axis in microvolts. The three waveform components of the early cortical evoked response, P1-N1-P2, are labeled. The vertical line at 0 ms denotes stimulus onset

We then explored the potential utility of using the hybrid electrophysiology paradigm to identify electrophysiologic features characteristic of visual processing in individuals with LFA in a pilot study with three participant groups. VEP waveforms recorded from electrode Oz in response to the checkerboard stimuli and averaged by group (LFA, HFA, TD) are shown in Fig. 1. The corresponding waveform latency and amplitude measurements (P1-N1-P2) are listed in Table 2. Individual waveforms are displayed by participant group in Supplementary Figures S1–3.

**Table 2:** Visual evoked potential measurements for the three participant groups. Mean peak latencies (ms) and amplitudes (μV) are shown for the early P1-N1-P2 waveform components; standard deviations are in parentheses

Group	P1		N1		P2	
	*Latency*	*Amplitude*	*Latency*	*Amplitude*	*Latency*	*Amplitude*
LFA	94.7 (±25.8)	3.34 (±1.79)	141.3 (±26.5)	-1.27 (±3.12)	221.3 (±28.0)	3.43 (±2.32)
HFA	82.0 (±22.4)	2.98 (±1.42)	116.8 (±30.2)	-2.44 (±2.66)	208.7 (±27.7)	5.02 (±2.59)
TD	78.7 (±8.2)	1.94 (±1.92)	115.3 (±16.1)	-1.95 (±3.46)	192.0 (±26.4)	5.23 (±2.19)

LFA: Low-Functioning Autism. HFA: High-Functioning Autism. TD: Typical Development

The waveform plots were inspected visually to identify features or trends differentiating the LFA group from the other two groups (Fig. 1). Two trends were identified. First, all three waveform components (P1-N1-P2) peaked consistently later for participants with LFA compared to participants with HFA and TD. Second, participants with LFA showed larger P1 amplitudes and smaller N1 and P2 amplitudes than the other two groups.

The P1 component peaked later and appeared larger on average for participants with LFA (94.7 ms ±25.8; 3.34 μV ±1.79) than for HFA (82.0 ms ±22.4; 2.98 μV ±1.42) and TD (78.7 ms ±8.2; 1.94 μV ±1.92). The average N1 response of participants with LFA also peaked later but was smaller (141.3 ms ±26.5; −1.27 μV ±3.12) than that of participants with HFA (116.8 ms ±30.2; −2.44 μV ±2.66) and TD (115.3 ms ±16.1; −1.95 μV ±3.46;). Similarly, the average P2 response of the LFA group was later and smaller (221.3 ± 28.0 ms; 3.43 ± 2.32 μV) compared to that of the HFA (208.7 ± 27.7 ms; 5.02 ± 2.59 μV) and TD (192.0 ± 26.4 ms; 5.23 ± 2.19 μV) groups. Across the three groups, a graded pattern was noted for the N1 and P2 components: Latencies were shortest and peak amplitudes were largest for the TD group, followed by the HFA group. Latencies were longest and peak amplitudes smallest for the LFA group.

In terms of within-group variability in VEP measurements, although standard deviations were generally largest for the two autism groups, standard deviations of P1 and N1 amplitudes for the TD group exceeded both the LFA and HFA groups.

### Overall spectral power distributions

**Fig. 2:**
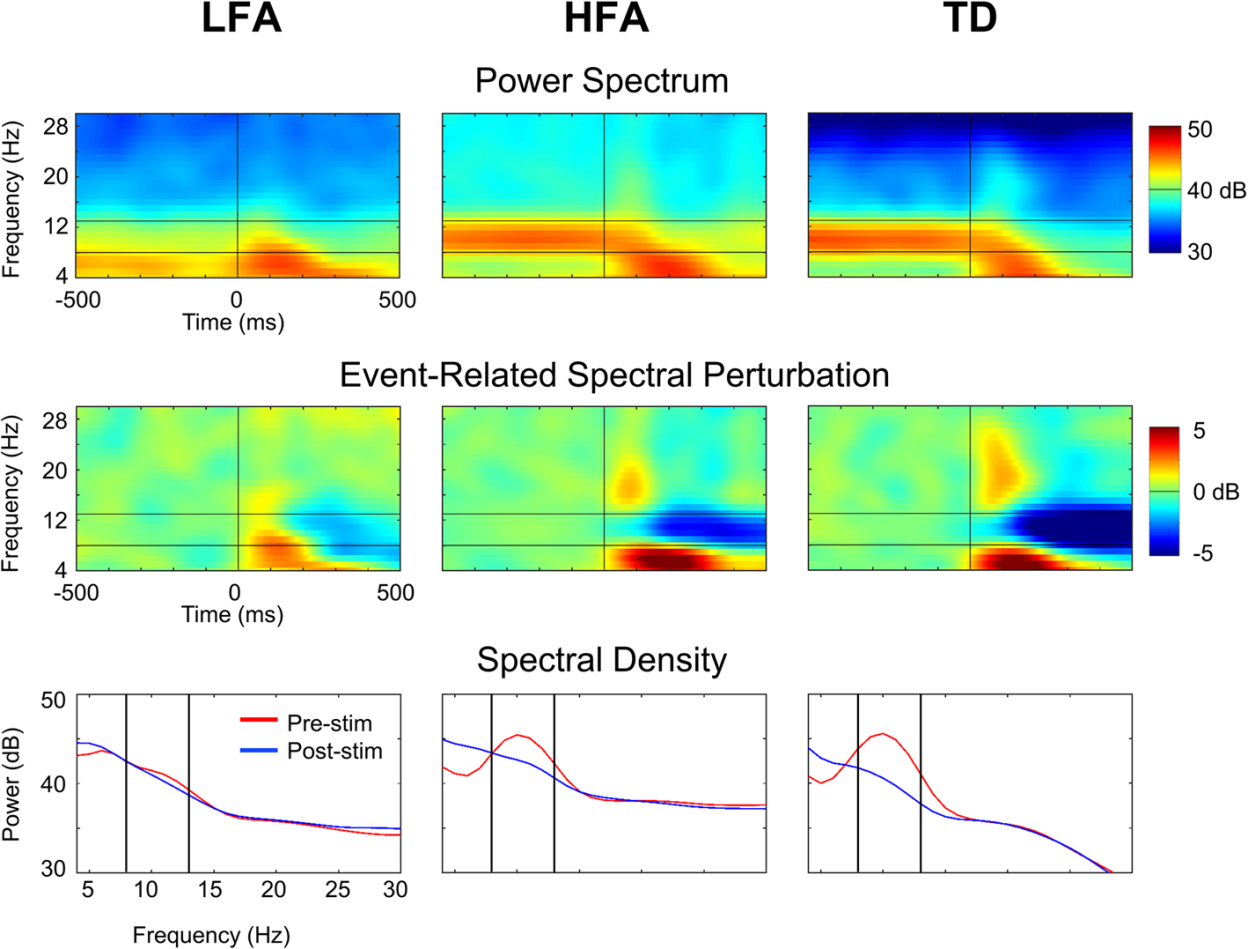
Power spectrum (top panel), event-related spectral perturbation (middle panel), and the spectral density (bottom panel) of three groups at Oz. The time window interval was [−500ms, 500 ms] with the checkerboard onset at 0 ms, and the frequency band analyzed was 4–30 Hz. Two horizontal lines in the top and middle panel indicate the alpha band (8–13 Hz). Two vertical lines in the spectral density plots indicate the alpha band

The mean overall distributions of spectral power between 4 and 30 Hz at electrode Oz, before and after presentation of the checkerboard stimulus, are shown for the three participant groups in Fig. 2 (top and bottom rows). Visual examination of the time-frequency plots revealed three observations of note. First, EEG spectral power (amplitude of modulation) was greatest in the theta-alpha range (4–13 Hz) across the HFA and TD groups for the pre- and poststimulus periods [−500, 500 ms]. In the LFA group, the spectral power was greatest in the theta band (4–8 Hz) for the pre- and poststimulus periods. Second, as shown in the spectral power density plots in Fig. 2 (bottom row), power in the alpha range (8–13 Hz) showed a unimodal peak in the prestimulus period that was largest for the TD group and decreased progressively from the HFA to the LFA groups, with the latter showing little to no observable alpha peak. Third, for the poststimulus period, all three groups showed increased theta activity (4–7 Hz), with HFA and TD groups showing more considerable theta change than the LFA group. A decrease in alpha power that began early in the poststimulus period (~50 ms) was also observed for the HFA and TD groups.

**Fig. 3:**
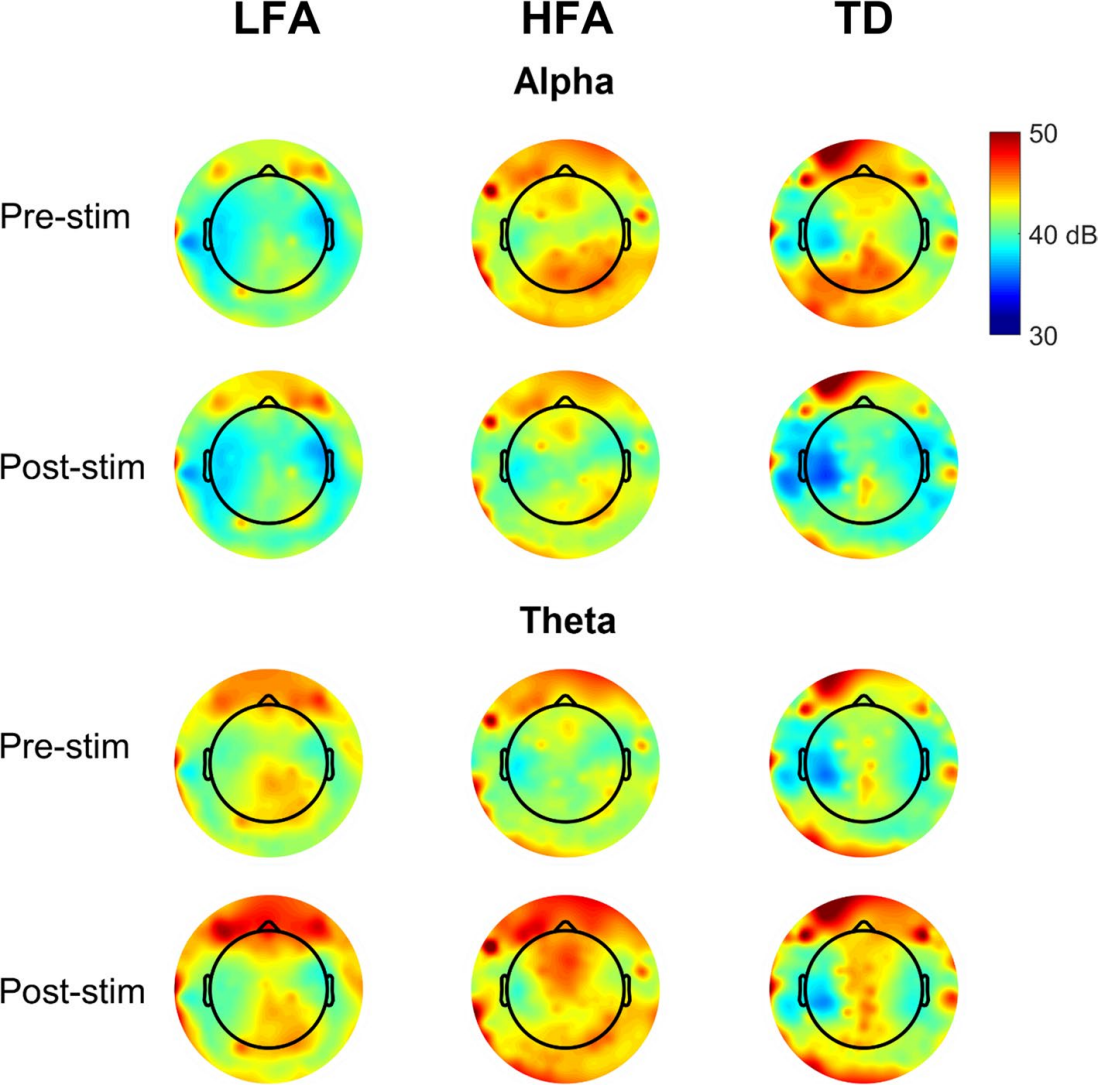
Topographical maps showing the distribution of prestimulus (−500 to 0 ms) and poststimulus (4 to 500 ms) spectral power in the alpha and theta bands for the three participant groups. LFA low-functioning autism, HFA high-functioning autism, TD typical development. Spectral power is color-coded as shown in the color bar with red representing the highest level of spectral power and blue representing the lowest level of spectral power

Topographical maps of the distribution of pre- and poststimulus alpha and theta spectral power across all electrode sites are shown for each of the three participant groups in Fig. 3. Examination of the topographical maps shows the gradient of prestimulus alpha power—from the weakest in the LFA group to strongest in the TD group—that is most prominent in the occipital region and also evident in the frontal region. Similarly, the absence of prestimulus increases in alpha activity, observed only for participants with LFA, does not appear to be limited to the occipital region but is evident across the cortex. Comparing pre- and poststimulus alpha power for the LFA group revealed no clear differences in activity level in contrast to both the HFA and TD groups, which both showed decreases in poststimulus alpha power relative to prestimulus levels. No group differences were observed in the distribution of pre- and poststimulus theta power across the three groups, with the largest increases in poststimulus theta observed in the occipital region for the HFA group.

### ERSP with visual stimulation

Mean within-group changes in poststimulus ERSP at electrode Oz are shown for each of the three participant groups in Fig. 2 (middle panel). Poststimulus enhancement of theta power was found to be statistically significant across all groups (*p* < 0.05). Poststimulus decreases in alpha power (*p* < 0.05) were observed for the HFA and TD groups. The LFA group showed small but significant decreases in alpha power. (See Supplementary Figure S4 for results of the permutation tests).

### Bootstrapped confidence intervals for pre- and poststimulus alpha and theta power

**Fig. 4:**
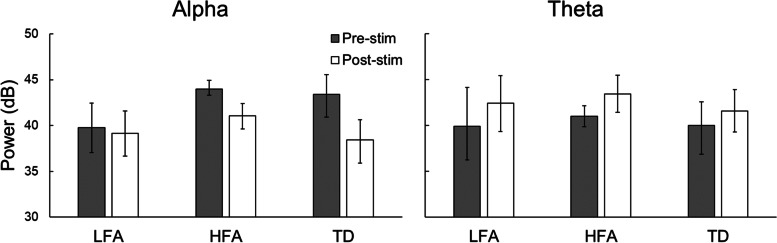
Bar graph showing average prestimulus (shaded gray) and poststimulus alpha (left panel) and theta (right panel) power for the three participant groups. Error bars indicate the 95% bias-corrected bootstrapped (*N*=2000) confidence intervals

The bias-corrected 95% bootstrapped confidence intervals (CI) for the mean pre- and poststimulus alpha and theta powers are shown in Fig. 4; the corresponding values are listed in Supplementary Table 1. Due to the relatively small sample size, statistical significance between group measures based on the confidence intervals cannot be confirmed. However, two results are noteworthy. The mean prestimulus alpha power in the LFA group (39.79 dB) is outside the 95% CIs of prestimulus alpha power of the HFA and TD groups. Also, while the HFA group had a similar level of prestimulus alpha power (43.98 dB) as the TD group (43.41 dB), the average poststimulus alpha power (41.06 dB) for the HFA group was much greater than the TD group (38.41 dB), suggesting larger alpha suppression in the TD group. The 95% CI for the poststimulus alpha of the HFA group does not include the mean of poststimulus alpha power of the TD group and vice versa. There are no notable differences in theta powers between LFA, HFA, and TD groups.

### Behavioral results

Although behavioral responses to the shape-discrimination stimuli were elicited mainly to encourage visual attention to the computer monitor for the randomly presented checkerboard stimuli, response accuracy and latency were analyzed for the three participant groups. The mean response time (RT) was fastest for the TD group (978.2 ms ±217.1) and slowest for the LFA group (1993.8 ms ±853.7), with intermediate RTs for the HFA group (1261.5 ms ±504.9). Response accuracy was > 98% for all participants, with the exception of two participants with LFA (P1 and P3) whose response accuracy was 83.7% and 48.2%, respectively.

## Discussion

Our results confirm the feasibility of using a hybrid visual paradigm to investigate visual evoked responses in individuals with LFA. We interleaved passively presented visual stimuli (checkerboards) among visual discrimination trials that participants had been trained to respond to, thereby maintaining their visual attention when the passive stimuli were presented. All participants with LFA completed the study, met adherence requirements, and demonstrated measurable visual evoked potentials. The high level of response accuracy that participants with LFA demonstrated on the behavioral discrimination trials coupled with review of the video recordings confirmed the visual discrimination trials helped to maintain visual attention to the computer screen for detection of the passively presented checkerboard stimuli. These results confirm the feasibility of including individuals with LFA in electrophysiology studies of visual processing across the autism spectrum.

Although no definitive conclusions can be drawn from the pilot study, results suggest potential group differences in evoked response patterns. Early cortical evoked responses recorded from individuals with LFA showed two distinct trends. First, waveform peak latencies were longer, on average, for the LFA group than for either the HFA or TD groups. Second, the amplitudes of the waveform components distinguished the LFA group from both the HFA and TD groups. Specifically, the P1 amplitude was larger in individuals with LFA, and the N1 and P2 amplitudes were consistently smaller. For the N1-P2 waveforms, we also observed a gradient pattern of latency and amplitude measurements across the three groups: the TD group had the shortest latencies and largest amplitudes on average, followed by the HFA group, while latencies were longer and amplitudes smaller for the LFA group. Although these observations are strictly exploratory and will require verification with larger groups, they underscore the utility of including individuals with LFA in autism studies of visual processing.

We also observed differences in the distribution of prestimulus and poststimulus alpha spectral power across the three groups. Prestimulus alpha power showed a gradient from weakest for the LFA group, followed by the HFA group, to the strongest for the TD group. Moreover, while both HFA and TD groups showed the expected decrease in poststimulus alpha power with visual stimulation, known as alpha suppression, this was largely absent in the LFA group. The lack of decreased poststimulus alpha activity observed in individuals with LFA likely reflects the absence of alpha activity in the prestimulus period and could be a potential marker of LFA.

These observations were supported by the bootstrapped CI results, suggesting that prestimulus alpha power could be an important electrophysiological marker for identifying individuals with LFA. Another interesting observation was that the bootstrapped CI for poststimulus alpha power for the HFA group did not include the mean poststimulus alpha power of the LFA and TD groups, while the LFA and TD groups’ bootstrapped CIs were similar. These findings suggest that the prestimulus alpha power and its modulation could be candidate electrophysiological markers of autism and its sub-groups, LFA and HFA. Based on the pilot study, larger-scale studies are planned to investigate potential electrophysiological response patterns that may be markers of atypical visual processing across the autism spectrum and could be useful for differentiating ASD subtypes.

## Conclusion

In summary, our results confirm the feasibility of using a modified visual paradigm to study evoked potentials in individuals with LFA and underscore the potential utility of including individuals with LFA in studies of visual processing to promote testing across the autism spectrum.

## Supplementary Information

**Additional file 1.**

**Additional file 2.**

**Additional file 3.**

**Additional file 4.**

**Additional file 5.**

**Additional file 6.**

## Data Availability

The data sets generated during the current study are not publicly available to comply with human subjects’ privacy protections, but they are available from the corresponding or first author on reasonable request.

## References

[1] American Psychiatric Association. Diagnostic and statistical manual of mental disorders: DSM-5. 5th ed. Arlington, VA: American Psychiatric Publishing Inc. 2013.

[2] Brown AC, Chouinard PA, Crewther SG. Vision research literature may not represent the full intellectual range of autism spectrum disorder. Front Hum Neurosci. 2017;11. 10.3389/fnhum.2017.00057 PMC5306295 28261072

[3] Jack A, Pelphrey KA (2017). Annual research review: understudied populations within the autism spectrum - current trends and future directions in neuroimaging research. J Child Psychol Psychiatry..

[4] Cantor DS, Thatcher RW, Hrybyk M, Kaye H (1986). Computerized EEG analyses of autistic children. J Autism Dev Disord..

[5] Kemner C, Verbaten MN, Cuperus JM, Camfferman G, Van Engeland H (1994). Visual and somatosensory event-related brain potentials in autistic children and three different control groups. Electroencephalogr Clin Neurophysiol..

[6] Bruneau N, Roux S, Adrien JL, Barthelemy C (1999). Auditory associative cortex dysfunction in children with autism: evidence from late auditory evoked potentials (N1 wave-T complex). Clin Neurophysiol..

[7] Marco EJ, Khatibi K, Hill SS, Siegel B, Arroyo MS, Dowling AF (2012). Children with autism show reduced somatosensory response: an MEG study. Autism Res..

[8] Lord C, Rutter M, DiLavore PC, Risi S, Gotham K, Bishop S (2012). Autism diagnostic observation schedule.

[9] Delorme A, Makeig S (2004). EEGLAB: an open source toolbox for analysis of single-trial EEG dynamics including independent component analysis. J Neurosci Methods..

[10] Groppe DM, Makeig S, Kutas M (2009). Identifying reliable independent components via split-half comparisons. Neuroimage..

[11] Benjamini Y, Hochberg Y (1995). Controlling the false discovery rate—a practical and powerful approach to multiple testing. J R Statist Soc B..

