# Supplementary figures and images for: Measuring visual electrophysiological responses in individuals with low-functioning autism: a feasibility and pilot study
Source: Pilot Feasibility Stud. 2022 Jan 14;8:7. doi: 10.1186/s40814-021-00960-7 (PMC8759181; doi:10.1186/s40814-021-00960-7)

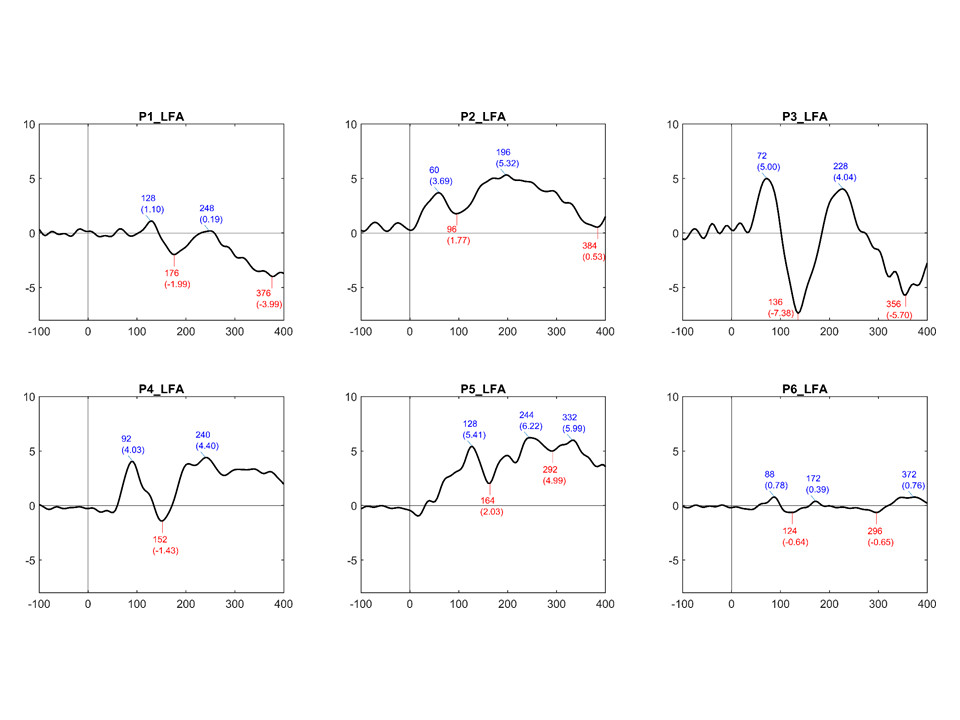

Supplement: Supplementary file 1 — Additional file 1. [file 40814_2021_960_MOESM1_ESM.tif]

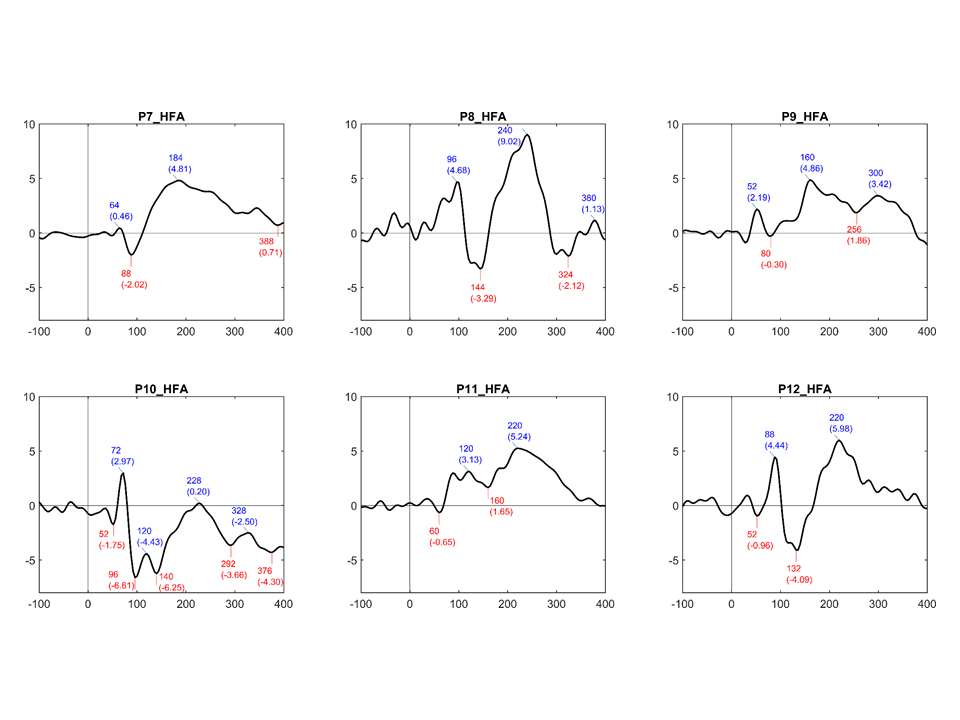

Supplement: Supplementary file 2 — Additional file 2. [file 40814_2021_960_MOESM2_ESM.tif]

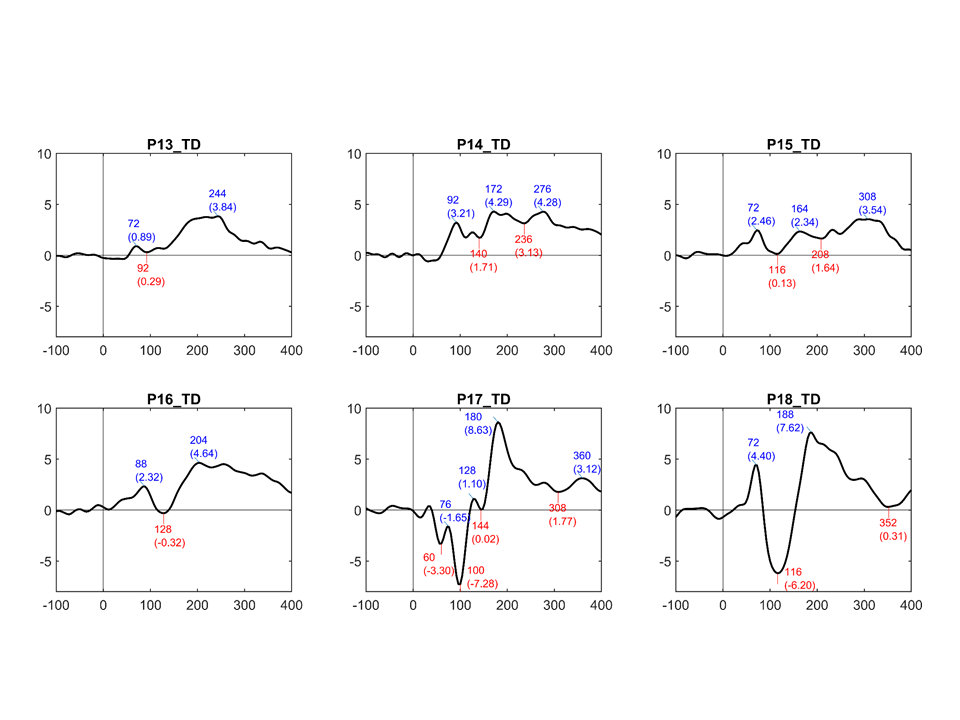

Supplement: Supplementary file 3 — Additional file 3. [file 40814_2021_960_MOESM3_ESM.tif]

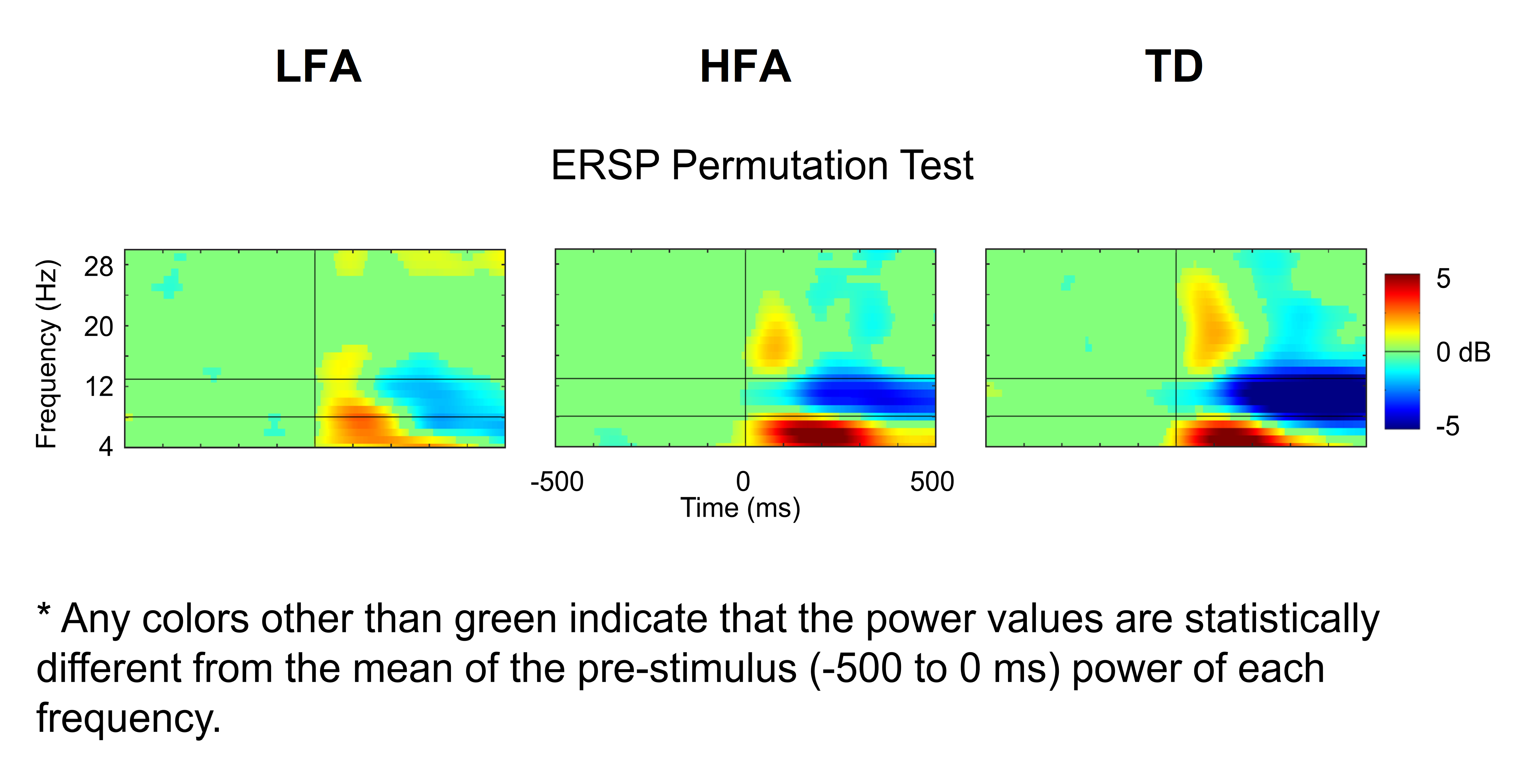

Supplement: Supplementary file 4 — Additional file 4. [file 40814_2021_960_MOESM4_ESM.tif]
