# Supplementary material for: Measuring visual electrophysiological responses in individuals with low-functioning autism: a feasibility and pilot study
Source: Pilot Feasibility Stud. 2022 Jan 14;8:7. doi: 10.1186/s40814-021-00960-7 (PMC8759181; doi:10.1186/s40814-021-00960-7)
Supplement: Supplementary file 5 — Additional file 5. [file 40814_2021_960_MOESM5_ESM.docx]

**Supplementary Table 1.** The mean (std dev) and bias-corrected 95% bootstrapping (N=2000) confidence intervals for pre- and poststimulus alpha and theta powers (dB).

| **Group** | **Frequency Bands** | **Time Windows** | **Mean Power  (Std. Dev.)** | **Bias-corrected 95% Bootstrapping CI** | |
| --- | --- | --- | --- | --- | --- |
|  |  |  |  | **Lower** | **Upper** |
| LFA | Alpha | Prestimulus | 39.79 (3.65) | 37.04 | 42.46 |
|  |  | Poststimulus | 39.17 (3.56) | 36.64 | 41.61 |
|  | Theta | Prestimulus | 39.93 (5.47) | 36.25 | 44.15 |
|  |  | Poststimulus | 42.43 (4.22) | 39.36 | 45.42 |
| HFA | Alpha | Prestimulus | 43.98 (1.05) | 43.31 | 44.91 |
|  |  | Poststimulus | 41.06 (2.21) | 39.62 | 42.39 |
|  | Theta | Prestimulus | 41.00 (1.80) | 39.86 | 42.14 |
|  |  | Poststimulus | 43.45 (3.09) | 41.41 | 45.46 |
| TD | Alpha | Prestimulus | 43.41 (2.66) | 40.90 | 45.57 |
|  |  | Poststimulus | 38.41 (3.00) | 35.89 | 40.64 |
|  | Theta | Prestimulus | 40.02 (3.34) | 36.87 | 42.58 |
|  |  | Poststimulus | 41.57 (3.06) | 39.30 | 43.91 |

LFA: Low-Functioning Autism; HFA: High-Functioning Autism; TD: Typical Development
